# Machine learning models for prediction of invasion Klebsiella pneumoniae liver abscess syndrome in diabetes mellitus: a singled centered retrospective study

**DOI:** 10.1186/s12879-023-08235-7

**Published:** 2023-05-04

**Authors:** Chengyi Feng, Jia Di, Shufang Jiang, Xuemei Li, Fei Hua

**Affiliations:** Department of Infection Control, The Third Affiliated Hospital of Soochow University, Changzhou, 213003 China

**Keywords:** Machine learning, Diabetes mellitus, Pyogenic liver abscess, Klebsiella pneumoniae, Prediction model

## Abstract

**Objective:**

This study aimed to develop and validate a machine learning algorithm-based model for predicting invasive Klebsiella pneumoniae liver abscess syndrome(IKPLAS) in diabetes mellitus and compare the performance of different models.

**Methods:**

The clinical signs and data on the admission of 213 diabetic patients with Klebsiella pneumoniae liver abscesses were collected as variables. The optimal feature variables were screened out, and then Artificial Neural Network, Support Vector Machine, Logistic Regression, Random Forest, K-Nearest Neighbor, Decision Tree, and XGBoost models were established. Finally, the model's prediction performance was evaluated by the ROC curve, sensitivity (recall), specificity, accuracy, precision, F1-score, Average Precision, calibration curve, and DCA curve.

**Results:**

Four features of hemoglobin, platelet, D-dimer, and SOFA score were screened by the recursive elimination method, and seven prediction models were established based on these variables. The AUC (0.969), F1-Score(0.737), Sensitivity(0.875) and AP(0.890) of the SVM model were the highest among the seven models. The KNN model showed the highest specificity (1.000). Except that the XGB and DT models over-estimates the occurrence of IKPLAS risk, the other models' calibration curves are a good fit with the actual observed results. Decision Curve Analysis showed that when the risk threshold was between 0.4 and 0.8, the net rate of intervention of the SVM model was significantly higher than that of other models. In the feature importance ranking, the SOFA score impacted the model significantly.

**Conclusion:**

An effective prediction model of invasion Klebsiella pneumoniae liver abscess syndrome in diabetes mellitus could be established by a machine learning algorithm, which had potential application value.

**Supplementary Information:**

The online version contains supplementary material available at 10.1186/s12879-023-08235-7

## Introduction

Pyogenic liver abscess is a purulent lesion caused by the invasion of pathogenic bacteria into the liver. The incidence of PLA varies slightly in various regions of the world and is increasing yearly [1]. The incidence rate in European and American countries was about (1.0 ~ 4.1)/100,000, the annual incidence rate in some Asian countries was (12 ~ 18)/100,000, and the annual incidence rate in mainland China was about (1.1 ~ 5.4)/100,000 [1–4]. Incidence is higher in males than females (3.3/100,000 vs. 1.3/100,000) [5]. Although the diagnosis and cure rates of PLA had improved significantly with the development of medical technology, the mortality rate was still around 10% [6]. In China and throughout the Asia–Pacific region, the primary pathogen of PLA is Klebsiella pneumoniae [7], which is prevalent in diabetic patients [4]. Diabetic patients with Klebsiella pneumoniae liver abscess are likelier to develop the invasive syndrome, IKPLAS [8]. IKPLAS refers to Klebsiella pneumoniae liver abscess with metastatic infection such as lung abscess, endophthalmitis, meningitis, necrotizing fasciitis, etc.IKPLAS has the characteristics of acute onset, rapid disease progression, and lack of specific clinical manifestations. If patients are not diagnosed and treated in time, the prognosis is generally poor [9]. Although there have been some studies on IKPLAS in the past, most of them are case reports [10–13], and there is no relevant literature report on its clinical prediction model.

Compared with traditional statistical methods, machine learning, as a branch of artificial intelligence, can analyze and obtain rules from existing data and continuously improve and build models based on algorithms and data [14]. Furthermore, it shows apparent advantages in clinical diagnosis and prognosis prediction [15, 16]. This study compared the performance of seven different machine learning methods in predicting the progression of the invasive Klebsiella pneumoniae liver abscess syndrome. Then, a model that can effectively identify high-risk patients is selected, which can help clinical decision-making and provide new perspectives for research in this field.

## Materials and methods

### Patients pre‑processing

This study included patients with diabetes and Klebsiella pneumoniae liver abscesses admitted to Changzhou First People's Hospital from January 1, 20,15 to December 31, 2021. The inclusion criteria were (1) Imaging showed liver abscess, and the puncture fluid or microbial blood culture was Klebsiella pneumoniae. (2) Diabetes diagnosis was based on the "Chinese Guidelines for the Prevention and Treatment of Type 2 Diabetes, 2020 Edition". The exclusion criteria were (1) Patients who died on admission. (2) Patients are automatically discharged or referred midway through. (3) Liver abscess secondary to primary or metastatic liver tumors. (4) Patients with abnormal coagulation function, platelet count, or dysfunction in the past. (5) The age is less than 18 years old. The primary observation was a diagnosis of IKPLAS during hospitalization. The diagnostic criteria of IKPLAS were liver abscess caused by Klebsiella pneumoniae and metastatic infection such as lung abscess, endophthalmitis, meningitis, necrotizing fasciitis, etc. The diagnosis of IKPLAS was judged by two physicians with senior professional titles in the clinic. Both physicians needed to be diagnosed with IKPLAS before establishing the diagnosis. Secondary observation indicators include general information (such as age, gender, comorbidities, etc.), the first laboratory (blood routine, liver and kidney function, etc.) and imaging (abdominal B-ultrasound) related indicators, treatment plans, etc. after admission. Among them, the medical history collection and routine blood test were collected on the day of admission, and the results of the first examination after admission by abdominal B-ultrasound, the treatment plan, and the prognosis were collected retrospectively after the patients were discharged from the hospital.

### Data pre-processing

Statistical analysis was performed using EmpowerStats software and Python 3.9, and the procalcitonin with too many missing values (number of missing values ≥ 30%) was deleted. Multiple imputations were performed for C-reactive protein, triglyceride, and cholesterol with a few missing values (number of missing values ≤ 30%) using the miceforest package in Python. Since different indicators are not comparable due to their different dimensions, we use the Z-score method to standardize continuous variables. The formula is:

$$z=\frac{\chi -u}{\sigma }$$

Where μ is the average of the continuous variable across all samples, and α is the standard deviation. The influence of dimensions on the data can be eliminated after data standardization. K-S-L test and Q-Q plot were used to test the normality of measurement data. The binary variables were described as counts, and percentages were evaluated using the Chi-square test or Fisher’s exact test. If the continuous variables conformed to a normal distribution, they were compared using a t-test and expressed as means ± SEM. For a non-normal distribution, the Mann–Whitney U test was used. *P* < 0.05 was considered statistically significant.

### Model training and evaluation

**Fig. 1:**
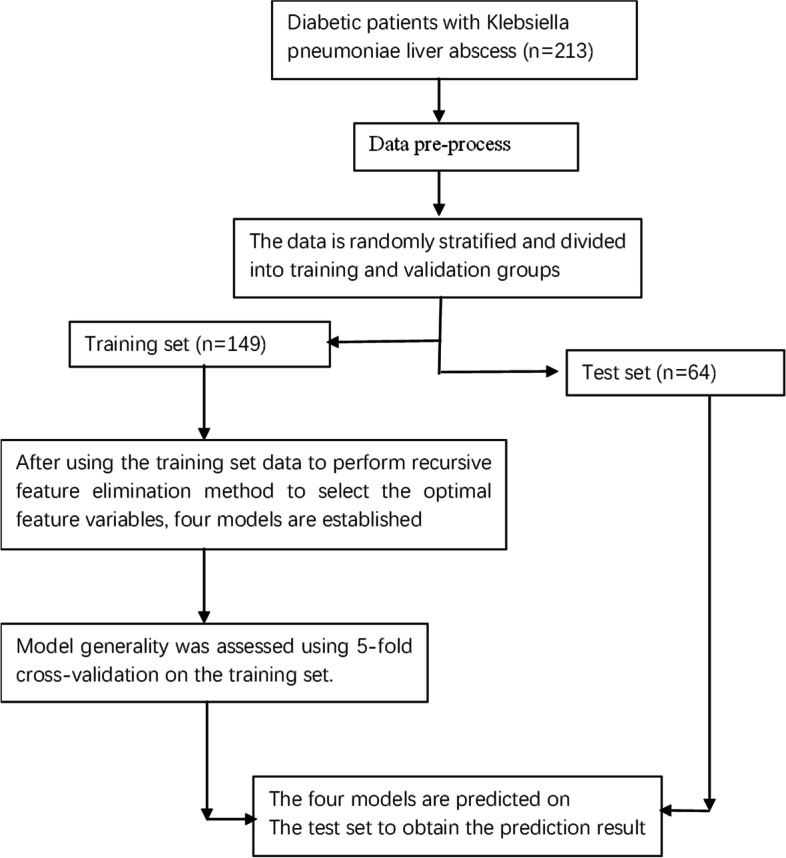
Overview of study design and model development

This research uses the python3.9 version, anaconda3 integrated development environment. Based on the train_test_split module, the parameter is set to test_size = 0.3, and the complete data is divided into a training set of 149 cases and a test set of 64 cases by stratified random sampling in a ratio of 7:3. This study used recursive feature elimination (RFE) for feature screening [17]. RFE can effectively eliminate the redundancy between features and select the optimal feature combination. It takes the prediction accuracy as the evaluation standard and eliminates the minimum relevant variables through each iteration. Then cross-validation is used to find the optimal number of features. In this study, random forest (RF) was used as the primary classifier for RFE, and feature selection was performed on the training set. The Scikit-learn python software package was used to build seven machine learning prediction models. The logistic regression model(LR) [18] was selected for the linear model. The Multilayer Perceptron (MLP) [19] model, also called artificial neural network (ANN), was chosen as an essential nonlinear prediction model. For the kernel-based model, Support Vector Machine (SVM) [20] with Gaussian kernel (RBF) was selected.For the decision tree approach, the random forest(RF) [21] model,the Decision Tree (DT) [22]model and the XGBoost(XGB) [23]model have also been used in clinical research. Finally, we chose a basic prediction model, the K-Nearest Neighbor algorithm (KNN) [24]. After the model was established, Bayesian optimization algorithm was used to find the maximum model Area Under Curve(AUC) value according to the Settings for parameter optimization. The specific optimized parameters were the C value of LR model, max_depth, min_samples_split, min_samples_leaf, min_weight_fraction_leaf of DT model, and max_depth, min_samples_leaf of LR model, n_estimators, max_features, max_depth, min_weight_fraction_leaf of RF model, n_estimators, max_leaves, max_depth, max_bin of XGB model, C-value and gamma of SVM model, hidden1, hidden2, learning_rate_int of ANN model and n_neighbors of KNN model.A fivefold cross-validation method was used to evaluate the model's generality in the training set. The model performance was evaluated using the test set, and the evaluation indicators were accuracy, precision, specificity, sensitivity (recall), F1-score, confusion matrix and AUC. A schematic overview of the study design and model development is depicted in Fig. 1.

## Results

### Patients and variables

**Fig. 2:**
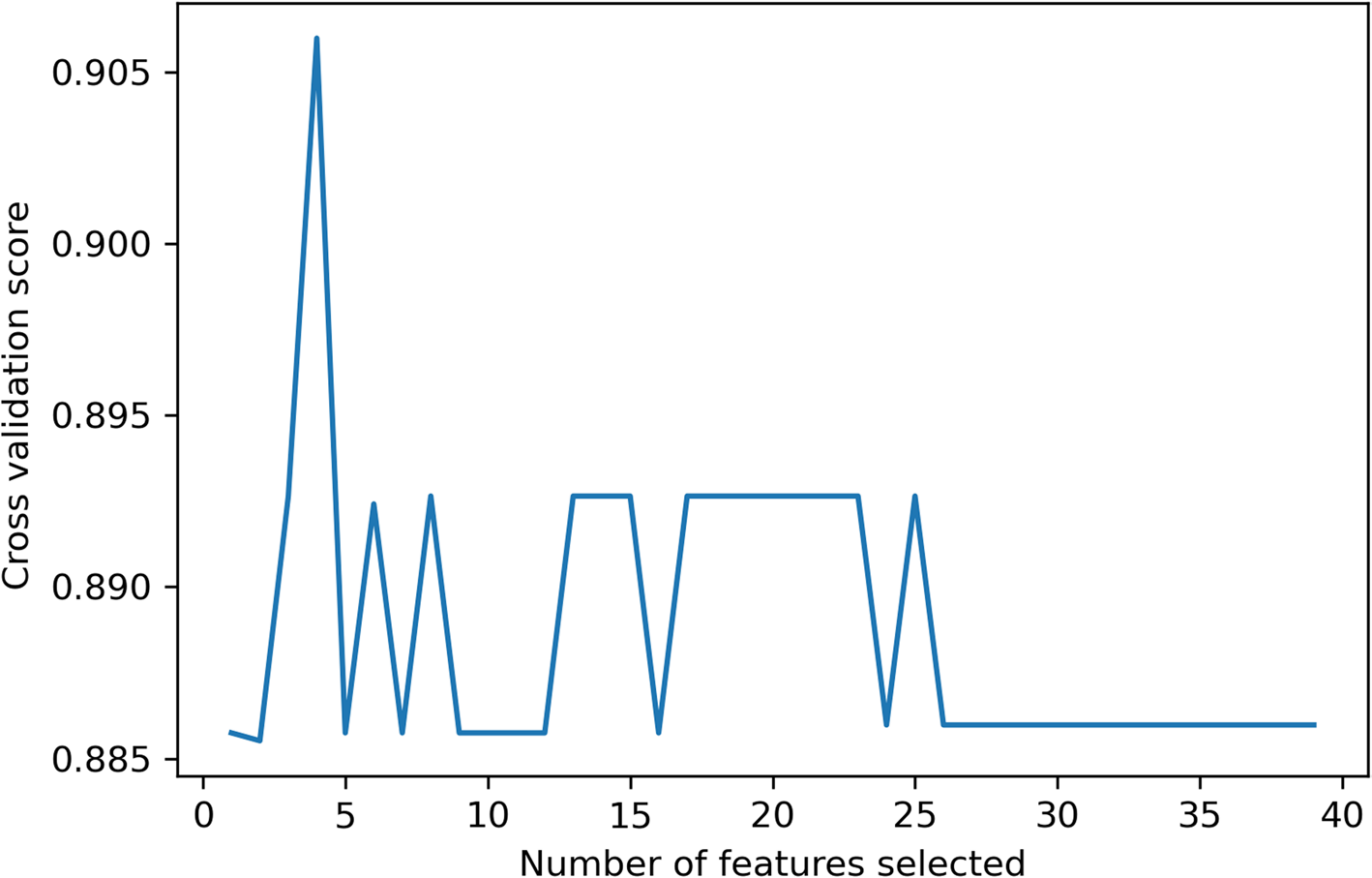
Recursive feature elimination method variable selection diagram

**Fig. 3:**
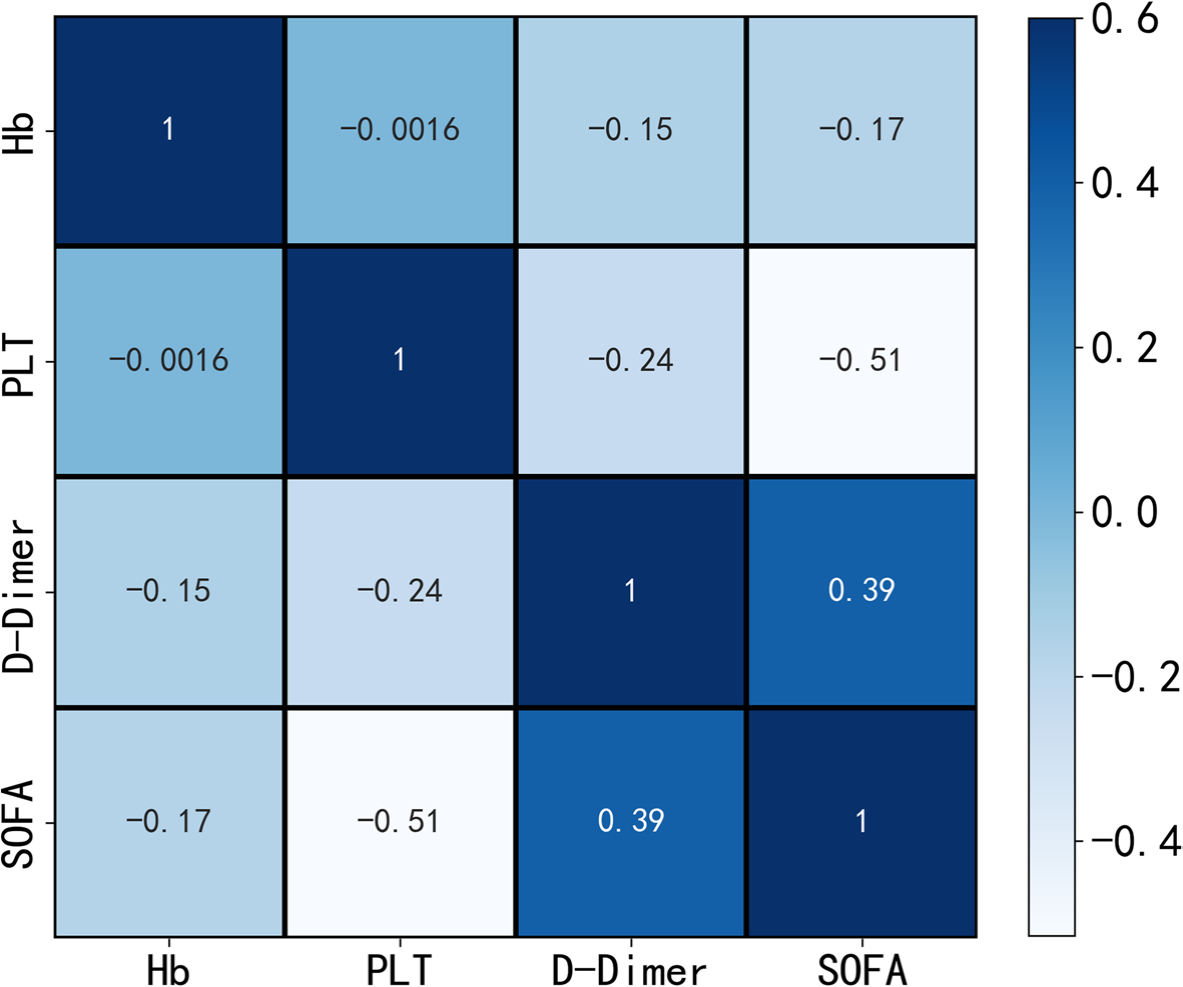
Spearman Correlation Analysis Heatmap

After screening by inclusion and exclusion criteria, 213 patients were included in this study, all in line with the diagnosis of type 2 diabetes mellitus and Klebsiella pneumoniae liver abscess. Patients were grouped by the occurrence of IKPLAS, with 25 cases progressing to IKPLAS as the IKPLAS group and 188 cases as the NIKPLAS group. There were 60 females and 153 males, as shown in Table 1. Through stratified random sampling, the data set was divided into the training set and test set. As shown in Table S1, there was no statistically significant difference between training set and test set(*P* ≥ 0.05). Clinical findings, Symptom at presentation, Admission data, and Radiologic findings in Table S1 will all be screened as variables. As shown in Fig. 2, When the number of feature variables is four, the recursive feature elimination method with random forest as classifier has the highest cross validation score. These four variables are hemoglobin, platelets, D-dimer, and SOFA score.Spearman correlation analysis was performed on these four features, as shown in Fig. 3, indicating no highly correlated redundant features.

**Table 1:** Baseline statistics for 213 patients Line 143

Characteristics	ALL(*N* = 213)	NIKLAS (*N* = 188)	IKLAS (*N* = 25)	*P*-value
**Clinical findings**				
AGE	61.5 ± 12.2	61.43 ± 12.05	61.92 ± 13.62	0.851
Gender				0.150
Female	60 (28.2%)	56 (29.79%)	4 (16.00%)	
Male	153 (71.8%)	132 (70.21%)	21 (84.00%)	
Smoke	30 (14.1%)	24 (12.77%)	6 (24.00%)	0.129
Alcohol	23 (10.8%)	18 (9.57%)	5 (20.00%)	0.115
DM(Year)	3.0 (2.0–10.0)	3.0 (2.0–9.2)	5.0 (2.0–10.0)	0.250
Underlying disease				
Biliary diseases	46 (21.6%)	39 (20.74%)	7 (28.00%)	0.408
CHD	19 (8.9%)	18 (9.57%)	1 (4.00%)	0.358
Liver diseases	16 (7.5%)	15 (7.98%)	1 (4.00%)	0.478
**Symptom at presentation**				
Body temperature	38.6 ± 1.1	38.62 ± 1.09	38.61 ± 1.01	0.962
Weakness	82 (38.5%)	62 (32.98%)	20 (80.00%)	< 0.001
Diarrhea	8 (3.8%)	5 (2.66%)	3 (12.00%)	0.021
Vomiting	22 (10.3%)	20 (10.64%)	2 (8.00%)	0.684
Abdominal pain	80 (37.6%)	72 (38.30%)	8 (32.00%)	0.541
Chills	91 (42.7%)	78 (41.49%)	13 (52.00%)	0.580
**Admission data**				
SBP	125.5 ± 18.0	125.18 ± 16.93	127.72 ± 25.23	0.510
DBP	75.4 ± 10.6	75.25 ± 9.87	76.92 ± 15.32	0.461
GLU	10.3 ± 3.6	10.03 ± 3.38	12.67 ± 4.17	< 0.001
WBC	11.6 (8.9–15.4)	11.6 (8.8–15.1)	12.5 (9.3–19.4)	0.055
NE	9.9 (7.2–13.4)	9.8 (7.1–13.1)	10.6 (7.8–17.9)	0.159
HB	116.0 (105.0–128.0)	117.0 (106.8–130.0)	106.0 (97.0–114.0)	< 0.001
PLT	191.0 (119.0–273.0)	205.0 (124.0–290.0)	125.0 (51.0–182.0)	0.003
ALT	57.0 (35.0–93.0)	57.0 (35.0–93.0)	58.0 (27.3–90.9)	0.509
AST	40.0 (25.7–78.2)	39.0 (25.6–73.2)	52.0 (26.0–113.0)	0.207
ALP	155.0 (103.0–240.0)	146.5 (103.0–240.0)	184.0 (133.0–239.0)	0.116
LDH	221.0 (176.0–299.0)	219.5 (176.8–289.2)	265.0 (170.0–343.0)	0.052
ALB	29.5 (26.7–33.2)	29.9 (27.2–33.5)	27.3 (24.1–29.9)	0.021
TBIL	12.9 (8.6–21.4)	12.6 (8.5–19.8)	15.8 (9.8–31.1)	0.013
DBIL	6.5 (4.2–10.9)	6.2 (4.0–10.4)	9.5 (5.6–13.5)	0.009
IBIL	5.7 (3.8–9.2)	5.7 (3.8–8.4)	5.7 (3.8–11.7)	0.209
BUN	5.0 (3.6–7.5)	4.9 (3.6–7.3)	5.8 (4.1–9.3)	0.082
TC	3.2 (2.6–3.9)	3.3 (2.7–3.9)	2.7 (2.2–3.1)	0.014
TG	1.4 (1.0–1.9)	1.4 (1.0–2.0)	1.3 (1.2–1.8)	0.755
CRP	114.0 (69.7–184.6)	112.5 (69.7–173.4)	163.0 (72.3–222.4)	0.019
PT	13.2 (12.4–14.1)	13.1 (12.3–13.9)	13.6 (13.0–15.3)	0.017
D.DIMER	2.5 (1.2–4.7)	2.2 (1.2–4.2)	4.4 (3.7–8.2)	< 0.001
MDRO	13 (6.1%)	10 (5.32%)	3 (12.00%)	0.190
SOFA	1.3 ± 2.3	0.87 ± 1.50	4.88 ± 3.66	< 0.001
**Radiologic findings**				
Abscess location				0.672
Right lobe	160 (75.1%)	140 (74.47%)	20 (80.00%)	
Left lobe	39 (18.3%)	36 (19.15%)	3 (12.00%)	
Both lobes	14 (6.6%)	12 (6.38%)	2 (8.00%)	
Abscess size (cm)	7.3 ± 2.5	7.41 ± 2.59	6.81 ± 2.04	0.271
No. of abscesses				0.151
Multiple	52 (24.4%)	43 (22.87%)	9 (36.00%)	
Solitary	161 (75.6%)	145 (77.13%)	16 (64.00%)	
**Treatment**				
Drainage mode				0.003
None	32 (15.0%)	23 (12.23%)	9 (36.00%)	
Catheterization	135 (63.4%)	120 (63.83%)	15 (60.00%)	
Puncture	40 (18.8%)	40 (21.28%)	0 (0.00%)	
Surgery	6 (2.8%)	5 (2.66%)	1 (4.00%)

### Tuning of parameters

**Fig. 4:**
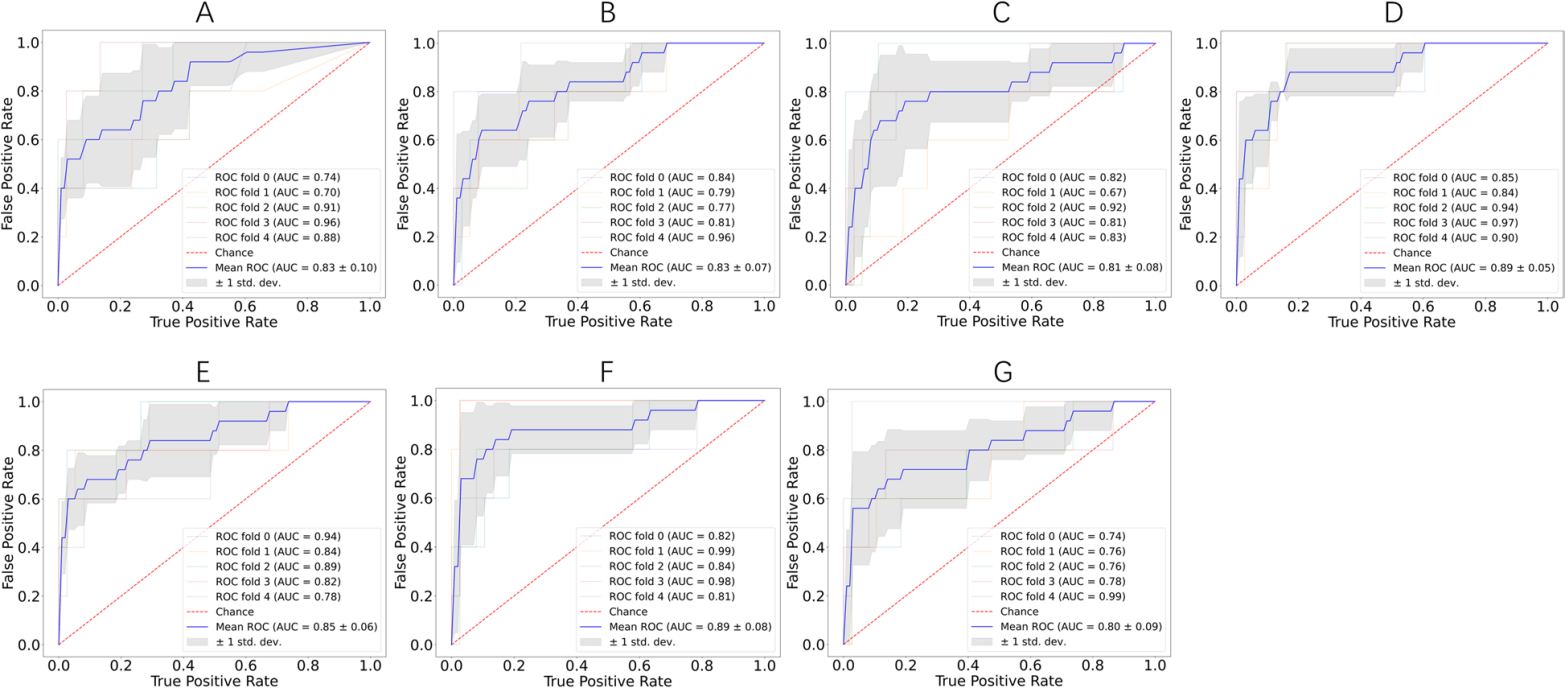
Five-fold cross-validation ROC curve for the training set. **A** ANN model. **B** DT model. **C** KNN model. **D** LR model. **E** RF model. **F** SVM model. **G** XGB model

The four variables selected from the training set were put into the machine learning classifier to construct the prediction model. Through Bayesian algorithm optimization, the parameters were adjusted with the average optimal AUC value, and the specific parameter Settings are shown in Table S2. The five-fold cross-validation ROC curve of the training set can be seen in Fig. 4, where it can be seen that the SVM model and LR model have better performance.

### Evaluation of prediction models

**Fig. 5:**
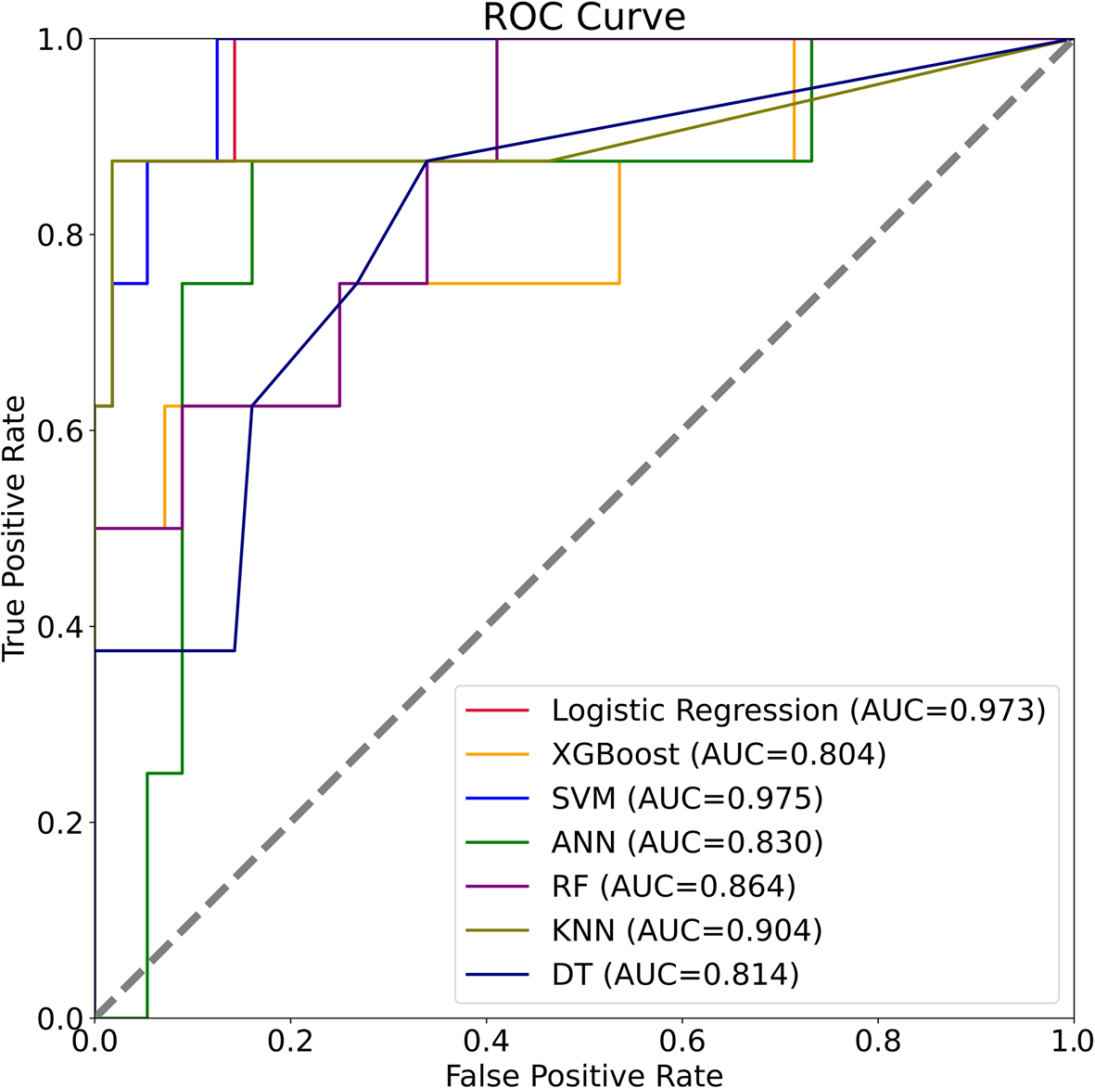
ROC curves of seven models in the test set

**Fig. 6:**
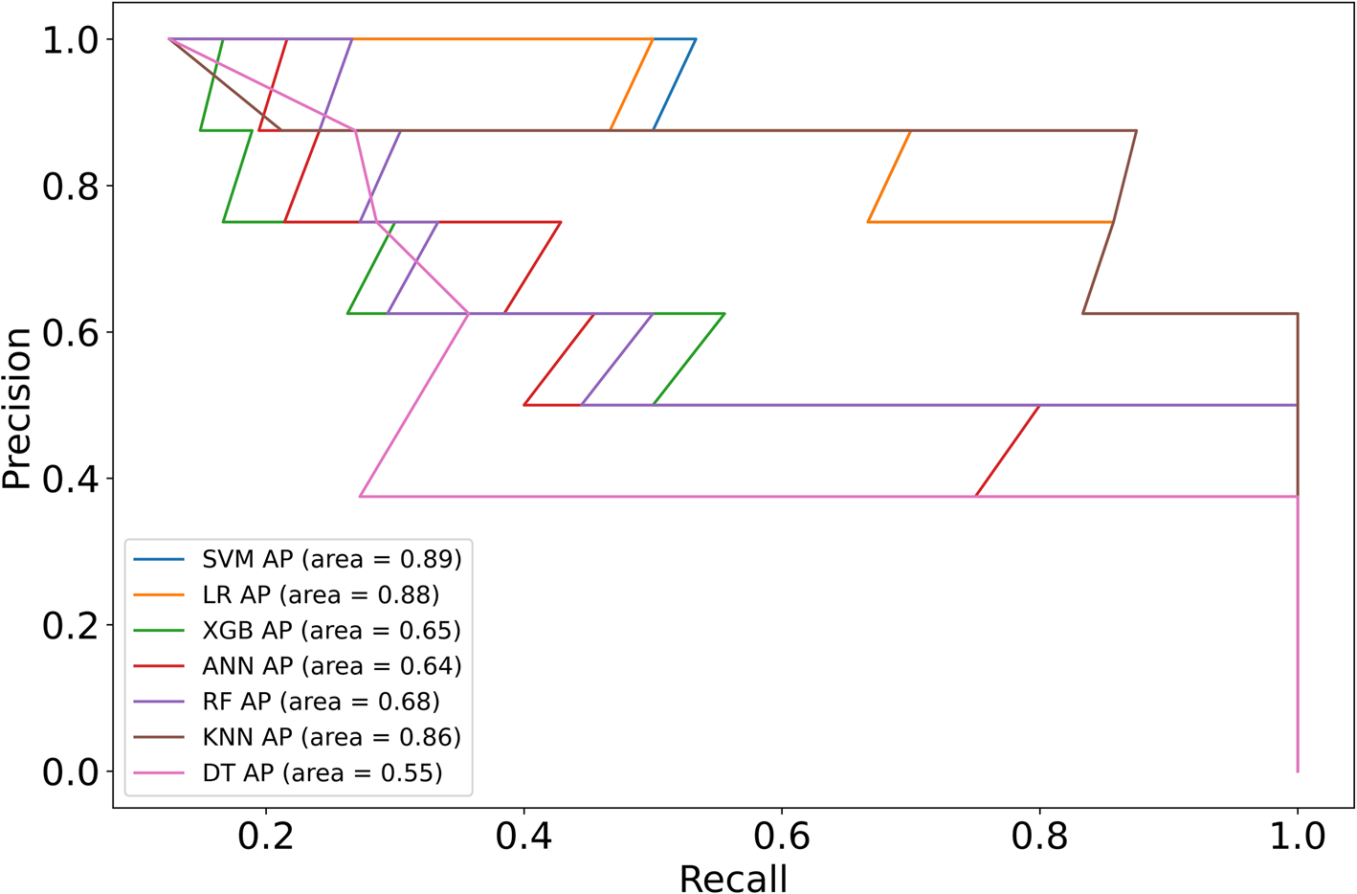
Precision Recall Curves for the seven models in the test set

The ROC curve of the test set can be seen in Fig. 5. The AUC values of most models are higher than 0,850, among which SVM (0.969) and LR (0.967) rank the top two, but the AUC values of XGB (0.799) and DT (0.800) are lower. Studies have shown that precision recall curve (PRC) has advantages over ROC in evaluating imbalanced datasets [25]. The dataset included in this study is also imbalanced, so PRC is also a valuable indicator. Figure 6 shows the PRC of the test set, and the Average Precision(AP) value was used as a criterion to evaluate the PR curve [26]. The APs of the LR,SVM models were all above 0.800. The confusion matrix was also calculated for all seven models (Table 2), and the DT model generated a large number of FPs (*n* = 19) during the prediction process, while the other models were relatively few. DT, LR, and SVM models produced the least FNs (*n* = 1), and the KNN model produced the least FPs (*n* = 0). Table 3 shows each model evaluation result's sensitivity (recall), specificity, accuracy, precision, f1, AP and AUC.

**Table 2:** Confusion matrices of 7 models

Confusion matrix	Actual	Prediction	
		Negative	Positive
SVM	Negative	52	4
	Positive	1	7
LR	Negative	49	7
	Positive	1	7
RF	Negative	53	3
	Positive	4	4
XGB	Negative	55	1
	Positive	4	4
ANN	Negative	51	5
	Positive	5	3
DT	Negative	37	19
	Positive	1	7
KNN	Negative	56	0
	Positive	6	2

**Table 3:** Performance summary in terms of sensitivity (recall), specificity, accuracy, precision,F1-score,AUC

Model	Sensitivity (recall)	Specificity	Accuracy	Precision	F1-Score	AP	AUC
SVM	0.875	0.929	0.922	0.636	0.737	0.890	0.969
LR	0.875	0.875	0.875	0.500	0.636	0.880	0.967
RF	0.500	0.946	0.891	0.571	0.533	0.680	0.879
XGB	0.500	0.982	0.922	0.800	0.615	0.650	0.799
ANN	0.375	0.911	0.844	0.375	0.375	0.640	0.897
DT	0.875	0.661	0.688	0.269	0.412	0.620	0.800
KNN	0.250	1.000	0.906	1.000	0.400	0.860	0.900

As shown in Table 3, there were significant performance differences between the models. The AUC (0.969), F1-Score(0.737) and AP(0.890) of the SVM model were the highest among the seven models, and the all-around performance was the best. At the same time, its sensitivity (0.875) is the highest and can effectively identify the occurrence of IKPLAS in the early stage. The KNN model had the best specificity (1.000) and could be used to reduce the occurrence of overdiagnosis and treatment.

**Fig. 7:**
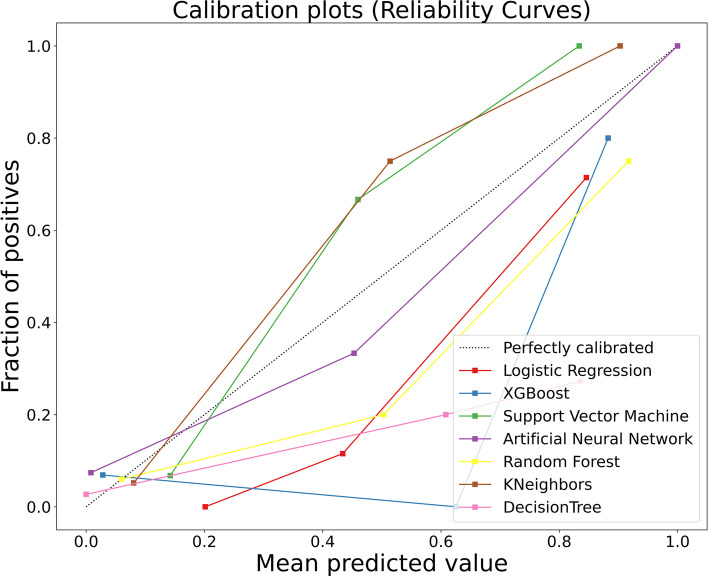
Seven machine learning model calibration curves

Figure 7 shows the calibration curves of the seven models. Except that the XGB and DT models over-estimates the occurrence of IKPLAS risk, the other models' calibration curves are a good fit with the actual observed results.

**Fig. 8:**
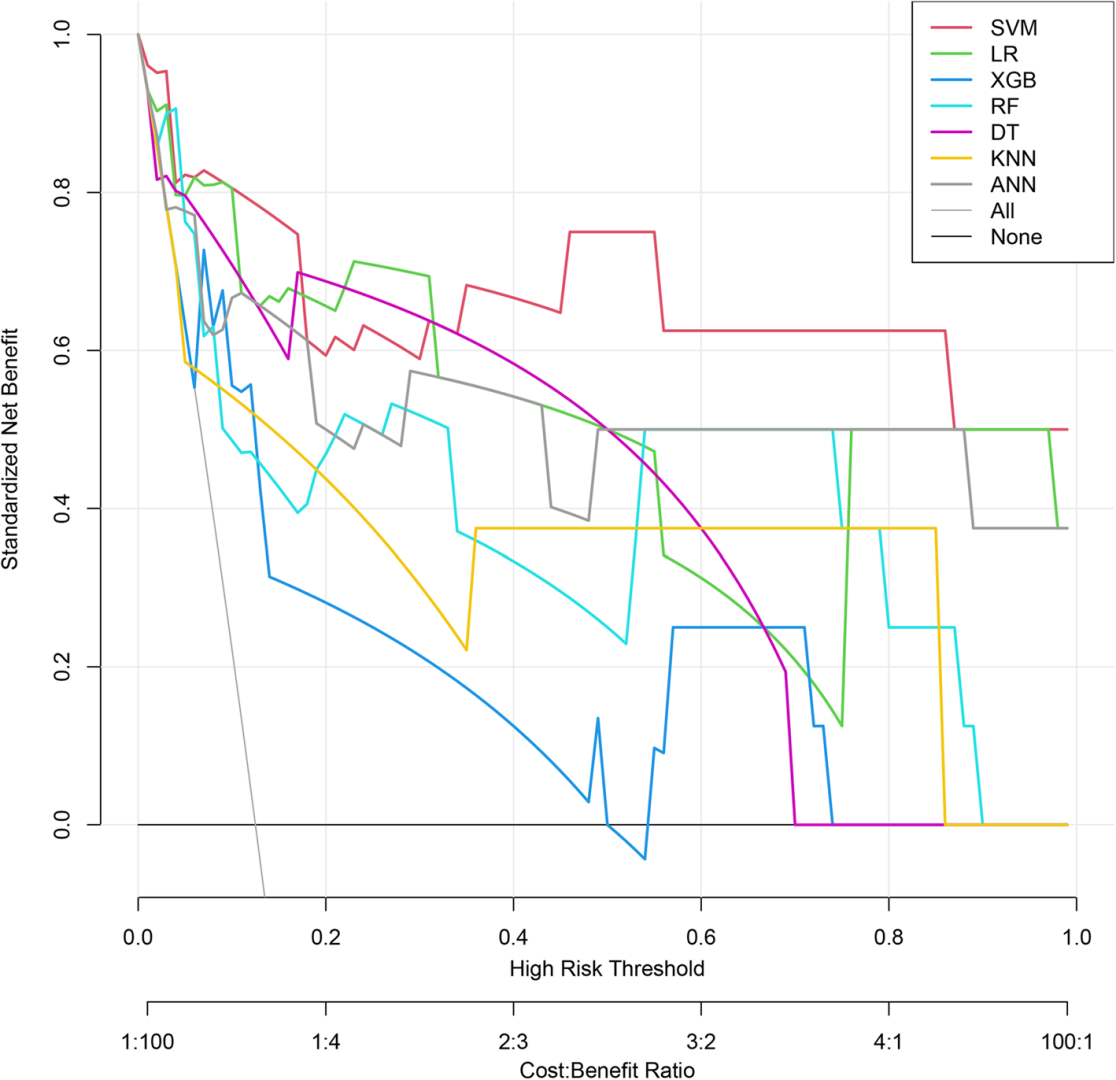
Decision curve analysis of seven machine learning models

Figure 8 shows the Decision Curve Analysis of the seven models, which was first proposed in 2006 and has been used for prognostic decision analysis in cancer [27] and other fields [23]. The DCA curve shows a model compared to the Net Benefit situation under different High-Risk Thresholds between the two strategies of intervention in all patients (ALL) and no intervention in all patients (NONE). As shown in Fig. 6, there is no significant difference in the benefits of treatment intervention based on SVM and LR model between the risk threshold of 0.0 and 0.4. However, when the risk threshold was between 0.4 and 0.8, the SVM model's net intervention rate was significantly higher than that of other models and the overall benefit rate was high. Model explanation.

To explain the output of our models, we used the SHapley Additive exPlanations(SHAP) algorithm to help us understand how a single feature affects the output of the models [28]. Its most significant advantage is that it can reflect the influence of the features in each sample, and it also shows the positive and negative effects of the influence. Each row represents a feature, sorted by feature importance from top to bottom. The abscissa is the SHAP value. A point represents a sample, and the color represents the eigenvalue (red for high, blue for low). The SVM prediction model with the best all-around performance was selected to interpret the feature importance.

**Fig. 9:**
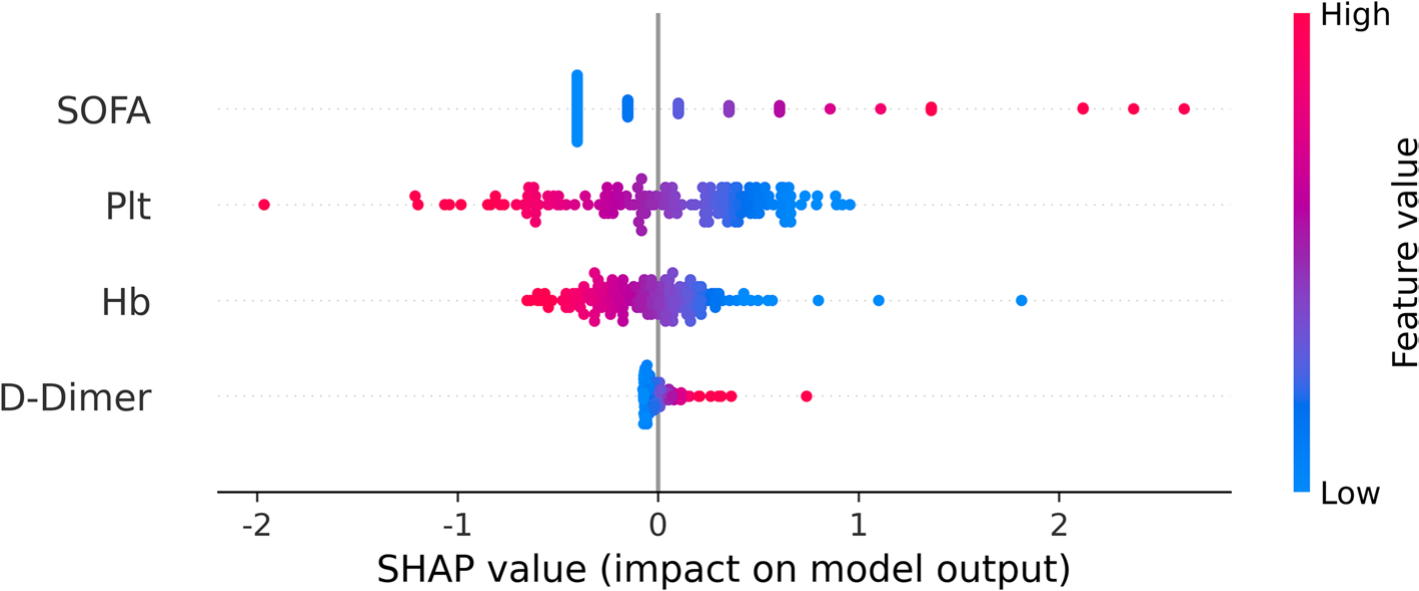
SHAP feature analysis of the SVM model

As shown in Fig. 9, the SOFA score ranks first in the feature importance of SVM model, and the higher the value, the higher the probability of the patient progressing to IKPLAS. Platelet and hemoglobin were the second and third most important predictors of the SVM model, and both were negatively correlated with the outcome. D-dimer ranked last and was positively associated with the risk of IKPLAS.

## Discussion

The high incidence of IKPLAS is mainly in the Asian population, which may be related to the fact that the Asian population is more likely to colonize the intestine with K1/K2 serotype Klebsiella pneumoniae [29, 30]. Diabetes is considered a significant risk factor for IKPLAS, and up to 63% of patients with a bacterial liver abscess in Taiwan have diabetes. This may be related to the impaired phagocytosis of K1/K2 Klebsiella pneumoniae in diabetic patients [31] and the more excellent vascular permeability in diabetic patients, which is conducive to bacterial invasion [11]. The above two serotypes of Klebsiella pneumoniae are also highly virulent Klebsiella pneumoniae, which show high viscosity in the String test [9]. Although the highly virulent Klebsiella pneumoniae is sensitive to most antibiotics, patients often have a poor prognosis if they are not recognized and treated early [32].

This study screened four characteristic variables: hemoglobin, platelets, D-dimer, and SOFA score. We interpreted the importance of the model characteristic variables by using the SHAP package, in which the SOFA score ranked first among all four models.

The SOFA score is a scoring system that measures the degree of impairment of significant organ function in patients with sepsis or suspected sepsis to determine prognosis [33]. Several studies have confirmed its predictive value in the prognosis of infected patients [34, 35]. This study also suggests that the SOFA score is a significant predictor of diabetes complicated by IKPLAS. As can be seen from the SHAP plot, the higher the SOFA score, the greater the risk of progression to IKPLAS. Although the pathogenesis of IKPLAS is currently unclear, the study by Chen-Guang Zhang et al. shows that most diabetic patients with IKPLAS are prone to sepsis [11]. Blood-borne transmission may be one of the more important ways.

In the feature importance ranking, platelets' influence on SVM model ranked second.Jai Hoon Yoon et al. showed that thrombocytopenia is an independent risk factor for invasive syndrome in diabetic patients with Klebsiella pneumoniae liver abscess [10]. This is also consistent with the conclusions about platelets in the SVM model established in this study. The mechanism of platelet reduction in diabetes combined with IKPLAS may be that when the body is infected, platelets are stimulated and activated to participate in the body's inflammatory response by inducing the expression of membrane proteins and the production of mediators and play the role of anti-infection and pathogen removal. Activated platelets produce and release pro-inflammatory, anti-inflammatory, chemokines, antimicrobial, and other mediators to regulate the body's innate immune or adaptive immune response [36]. The interaction between platelets and pathogens or their products, endothelial cells, and immune cells promotes endothelial cell damage and leukocyte activation. As a result, the adhesion of platelets to it is enhanced, platelets are continuously activated in the circulation, and the body continuously produces anti-platelet antibodies and macrophage-colony stimulating factors, which accelerates the destruction and consumption of platelets [37].

The SHAP plot shows that hemoglobin is the third most important characteristic variable after the SOFA score, and the lower its value, the higher the risk of progression to IKPLAS. It has been shown that hemoglobin can be an indicator to assess the severity of the disease in infected patients, probably due to a systemic inflammatory response leading to decreased erythropoiesis, increased destruction of erythrocytes due to hemolysis, and hemorrhage, which leads to a reduced ability of blood to transport oxygen and carbon dioxide and insufficient oxygen supply to the body, resulting in multi-organ damage [38].

D-dimer is a specific molecular marker for secondary hyperfibrinolysis in vivo and is an effective indicator to reflect the coagulation state of the body. The coagulation and fibrinolytic systems are usually closely linked to the development of inflammation. Infection can lead to damage of vascular endothelial cells and alveolar epithelial cells, which stimulates the coagulation system, resulting in impairment of coagulation function and abnormal coagulation indexes in patients, further aggravated by elevated D-dimer along with infection [39, 40]. The above two promote each other, forming a vicious circle. The autoimmune function of diabetic patients is weakened, and the inflammatory response is enhanced after infection. Patients with diabetes complicated with IKPLAS can have noticeable D-dimer changes in the early stage. In the SVM model, D-dimer was positively associated with the risk of developing diabetes with IKPLAS, which is consistent with the above findings.

In the field of IKPLAS, more studies are focused on the risk factors of IKPLAS.The study by Shixiao Li et al. [41] showed that patients with IKPLAS were more likely to develop chronic renal insufficiency, thrombocytopenia, and increased total bilirubin than patients with non-IKPLAS. Hairui Wang et al. [42]. A logistic regression prediction model was used to predict the incidence of IKPLAS by incorporating clinical and CT features, with an AUC value of 0.842 in the validation set, and did not compare other prediction models.Unlike many studies, we first used seven machine learning models for prediction. Through parameter adjustment and verification, the SVM model with the best performance was selected, with an AUC value of 0.969 and an AP value of 0.890, indicating that it was a reliable IKPLAS prediction model. At the same time, the variables included in this model are clinical indicators, which are easy to collect and can be used by clinicians to conveniently judge the possibility of IKPLAS in patients with diabetes mellitus complicated with Klebsiella pneumoniae liver abscess.

Machine learning algorithms can build complex models that perform satisfactorily enough when the amount of data is sufficient. However, in specific applications, the amount of data is often insufficient, so it is essential to analyze these machine learning algorithms and obtain good results with relatively small sample sizes. In this study, the Power analysis was satisfied by calculating a power value of > 0.80, although we only used a small data set of 213 patients. The main reason for the excellent performance of the SVM model in this study is that it is a nonlinear learner that is more suitable for small samples, can ideally separate samples, and has better generalization.

There are still some limitations in this study. First, this is a single-center regression study, and some potential biases cannot be avoided. Secondly, for machine learning, the sample size of this study is insufficient. In order to further improve the accuracy of the model, we will collect more clinical data and further optimize the parameters.

## Conclusion

In this study, we established and compared seven models to compare the performance of predicting the progression of diabetes with Klebsiella pneumoniae to IKPLAS and found that the SVM model had the highest overall predictive power. We also found that SOFA score, platelets, hemoglobin, and D-dimer significantly affected the model's predictions. In the future, we will expand the dataset to improve further the model's accuracy and better plan diagnosis and treatment for clinicians.

## Supplementary Information

**Additional file 1: Table S1.** Characteristics of patients in the training and test sets.

**Additional file 2:** **Table S2.** Tuning parameters of the predictive models.

## Data Availability

The datasets used and analyzed during the current study are available from the corresponding author on reasonable request.
